# Supplementary material for: Regenerating hair cells in vestibular sensory epithelia from humans
Source: eLife. 2018 Jul 18;7:e34817. doi: 10.7554/eLife.34817 (PMC6078492; doi:10.7554/eLife.34817)
Supplement: Supplementary file 1. — The left column lists gene names followed by gene descriptions. AB T is the average transcript abundance in the Atoh1 treated samples (as FPKMs). AB C is the average abundance in the control samples. LOG2 is the log base2 fold change of Atoh1 transfected compared to the controls. REFERENCE indicates the published results showing supportive evidence that the given gene is a putative hair cell marker (1) Cai et al. (2015); (2) Scheffer et al., 2015; (3) Shin et al. (2013). Names in bold are genes that show a > 2 fold change in expression (and p<0.05) across all three comparisons (they are part of the 441 differentially expressed genes, see materials and methods and results). Genes with an asterisk (*) are putative Atoh1 downstream targets based on Groves et al publication. Genes in plain text and above the thick bottom line show statistically significant upregulation in all comparisons, but only pass > 2 fold in at least one comparison. Genes below the thick bottom line do not pass any of the above significance thresholds and show at least upregulation in all comparisons. [file elife-34817-supp1.docx]

**Supplementary File 1: Known and putative hair cell markers that exhibit upregulation in gene expression upon Atoh1 transduction into human sensory epithelia.**

The left column lists gene names followed by gene descriptions. AB T is the average transcript abundance in the Atoh1 treated samples (as FPKMs). AB C is the average abundance in the control samples. LOG2 is the log base2 fold change of Atoh1 transfected compared to the controls. REFERENCE indicates the published results showing supportive evidence that the given gene is a putative hair cell marker 1: Cai et al., 2015; 2 Scheffer et al. 2015; 3: Shin et al., 2013. Names in bold are genes that show a >2-fold change in expression (and p<0.05) across all three comparisons (they are part of the 441 differentially expressed genes, see materials and methods and results). Genes with an asterisk (*) are putative Atoh1 downstream targets based on Groves et al publication. Genes in plain text and above the thick bottom line show statistically significant upregulation in all comparisons, but only pass >2-fold in at least one comparison. Genes below the thick bottom line do not pass any of the above significance thresholds and show at least upregulation in all comparisons.

| **GENE** | **DESCRIPTION** | **AB T** | **AB C** | **LOG2** | **REFERENCE** |
| --- | --- | --- | --- | --- | --- |
| **ACTC1** | **actin, alpha, cardiac muscle 1** | **2.5** | **0.1** | **4.5** | [1] |
| **ATOH1** | **atonal bHLH transcription factor 1** | **37.8** | **0.3** | **7.4** | [1],[2] |
| **CCDC60** | **coiled-coil domain containing 60** | **0.5** | **0.1** | **2.1** | [2] |
| **CES2** | **carboxylesterase 2** | **33.8** | **12.5** | **1.5** | [2] |
| **ENO2** | **enolase 2** | **334.9** | **74.6** | **2.2** | [2] |
| **EPS8L2*** | **EPS8 like 2** | **77.8** | **18.4** | **2.4** | [1],[2],[3] |
| **GPI** | **glucose-6-phosphate isomerase** | **185.7** | **58.8** | **1.7** | [3] |
| **HIST3H2A** | **histone cluster 3 H2A** | **30.9** | **9.0** | **1.9** | [1] |
| **JAG2*** | **jagged 2** | **13.0** | **3.1** | **2.3** | [1],[2] |
| **OBSCN*** | **obscurin, cytoskeletal calmodulin and titin-interacting RhoGEF** | **20.3** | **5.8** | **2.1** | [1] |
| **ODF3B** | **outer dense fiber of sperm tails 3B** | **6.8** | **2.0** | **2.1** | [1] |
| **PFKL** | **phosphofructokinase, liver type** | **94.8** | **38.2** | **1.3** | [3] |
| **PKN3*** | **protein kinase N3** | **5.2** | **2.1** | **1.4** | [1] |
| **POU4F3*** | **POU class 4 homeobox 3** | **3.4** | **0.8** | **2.4** | [1],[2] |
| **RABL2A** | **RAB, member of RAS oncogene family-like 2A** | **9.6** | **4.3** | **1.2** | [2] |
| **RAX** | **retina and anterior neural fold homeobox** | **0.7** | **0.1** | **2.6** | [1] |
| **SYT7** | **synaptotagmin 7** | **3.3** | **1.1** | **1.9** | [2] |
| **SYTL1** | **synaptotagmin like 1** | **8.8** | **2.6** | **3.2** | [1] |
| **TAS1R1** | **taste 1 receptor member 1** | **2.5** | **0.4** | **2.9** | [1],[2] |
| **UBA7** | **ubiquitin like modifier activating enzyme 7** | **46.1** | **8.4** | **2.8** | [3] |
| **UBXN11** | **UBX domain protein 11** | **18.1** | **7.6** | **1.3** | [2] |
| **UNC5A*** | **unc-5 netrin receptor A** | **5.0** | **0.4** | **4.8** | [1] |
| TTC21A* | tetratricopeptide repeat domain 21A | **6.0** | **3.2** | **1.6** | [1],[2] |
| TJP3* | tight junction protein 3 | **17.2** | **6.4** | **3.2** | [1] |
| RHPN1 | rhophilin Rho GTPase binding protein 1 | **22.7** | **6.4** | **2.5** | [2} |
| TACSTD2 | tumor-associated calcium signal transducer 2 | **211.5** | **95.9** | **2.0** | [1] |
| SALL1* | spalt like transcription factor 1 | **9.7** | **3.9** | **1.6** | [1],[2] |
| THOP1 | thimet oligopeptidase 1 | **38.3** | **18.9** | **1.1** | [3] |
| RAB11B | RAB11B, member RAS oncogene family | **80.4** | **41.9** | **1.0** | [3] |
| SPTAN1 | spectrin alpha, non-erythrocytic 1 | **118.0** | **66.4** | **0.8** | [3] |
| TMEM184A | transmembrane protein 184A | **8.5** | **2.7** | **3.0** | [1],[2] |
| HRASLS | HRAS like suppressor | **6.9** | **2.6** | **1.9** | [2] |
| UCKL1 | uridine-cytidine kinase 1 like 1 | **34.0** | **18.1** | **1.8** | [2] |
| APBB3 | amyloid beta precursor protein binding family B member 3 | **18.7** | **11.8** | **1.1** | [2] |
| MYO7A* | myosin VIIA | **9.9** | **1.5** | **3.2** | [1],[2],[3] |
| GFI1* | growth factor independent 1 transcriptional repressor | **1.7** | **1.0** | **0.8** | [1],[2] |
| NONO | non-POU domain containing, octamer-binding | **94.0** | **61.1** | **0.6** | [3] |
| RSPH9 | radial spoke head 9 homolog | **2.6** | **1.7** | **0.7** | [2] |
| TOLLIP | toll interacting protein | **43.6** | **33.6** | **0.5** | [3] |
| PARP6 | poly(ADP-ribose) polymerase family member 6 | **77.3** | **56.6** | **0.5** | [2] |
| MDH2 | malate dehydrogenase 2 | **113.1** | **75.8** | **0.7** | [3] |
| PPM1J | protein phosphatase, Mg2+/Mn2+ dependent 1J | **5.4** | **2.0** | **2.0** | [1] |
| CNDP2 | CNDP dipeptidase 2 (metallopeptidase M20 family) | **135.7** | **81.0** | **1.1** | [3] |
| LRRC27 | leucine rich repeat containing 27 | **6.3** | **4.3** | **1.2** | [2] |
| CNNM2 | cyclin and CBS domain divalent metal cation transport mediator 2 | **8.1** | **5.0** | **1.0** | [2] |
| GAA | glucosidase alpha, acid | **77.4** | **30.6** | **1.9** | [3] |
| CCDC96 | coiled-coil domain containing 96 | **1.7** | **1.0** | **0.9** | [1],[2] |
| CHRNA1 | cholinergic receptor nicotinic alpha 1 subunit | **11.2** | **2.8** | **4.3** | [1],[2] |
| MYO15A | myosin XVA | **7.6** | **2.4** | **2.1** | [1],[2],[3] |
| FSCN2 | fascin actin-bundling protein 2, retinal | **2.7** | **1.2** | **1.4** | [1],[2],[3] |
| NDUFV1 | NADH:ubiquinone oxidoreductase core subunit V1 | **108.1** | **67.1** | **1.2** | [3] |
| ANKRD24 | ankyrin repeat domain 24 | **3.9** | **1.8** | **1.3** | [2],[3] |
| MARVELD3 | MARVEL domain containing 3 | **16.2** | **4.7** | **2.9** | [1] |
| IMPDH1 | inosine monophosphate dehydrogenase 1 | **20.6** | **10.7** | **1.2** | [2] |
| SULT4A1* | sulfotransferase family 4A member 1 | **0.8** | **0.2** | **2.4** | [1],[2] |
| SLC7A4 | solute carrier family 7 member 4 | **1.9** | **1.0** | **1.7** | [2] |
| LLGL2* | LLGL2, scribble cell polarity complex component | **51.8** | **26.5** | **2.5** | [1] |
| PLA2G16* | phospholipase A2 group XVI | **85.1** | **63.7** | **0.6** | [1] |
| IGFBPL1 | insulin like growth factor binding protein like 1 | **2.1** | **0.6** | **3.4** | [1] |
| CGN | cingulin | **28.0** | **11.9** | **2.4** | [2] |
| CTBP2 | C-terminal binding protein 2 | **27.9** | **20.9** | **0.6** | [3] |
| CCDC146 | coiled-coil domain containing 146 | **3.7** | **2.3** | **1.3** | [1] |
| GLB1 | galactosidase beta 1 | **62.8** | **40.7** | **0.9** | [3] |
| FUT1 | fucosyltransferase 1 (H blood group) | **2.9** | **1.1** | **2.0** | [1] |
| NPEPPS | aminopeptidase puromycin sensitive | **78.4** | **53.9** | **0.7** | [3] |
| MRPS6 | mitochondrial ribosomal protein S6 | **512.2** | **228.0** | **1.3** | [2] |
| SPINT1* | serine peptidase inhibitor, Kunitz type 1 | **196.0** | **62.7** | **2.6** | [1],[2] |
| NPEPL1 | aminopeptidase-like 1 | **13.0** | **8.5** | **1.1** | [3] |
| TWF2 | twinfilin actin binding protein 2 | **37.1** | **19.7** | **1.0** | [2],[3] |
| SH3GLB2 | SH3 domain containing GRB2 like endophilin B2 | **57.5** | **34.8** | **0.8** | [3] |
| PRDX6 | peroxiredoxin 6 | **180.4** | **137.3** | **0.4** | [3] |
| TMEM183A | transmembrane protein 183A | **14.5** | **10.6** | **0.7** | [2] |
| CDH1* | cadherin 1 | **76.0** | **22.6** | **2.9** | [1] |
| RASSF4* | Ras association domain family member 4 | **24.5** | **10.2** | **1.6** | [1] |
| TTLL3 | tubulin tyrosine ligase like 3 | **18.7** | **13.2** | **0.6** | [2] |
| PDZD7* | PDZ domain containing 7 | **3.3** | **1.0** | **2.7** | [1] |
| SPTBN1 | spectrin beta, non-erythrocytic 1 | **136.9** | **81.5** | **0.9** | [3] |
| RGS11 | regulator of G-protein signaling 11 | **4.5** | **2.8** | **1.2** | [2] |
| KCNF1* | potassium voltage-gated channel modifier subfamily F member 1 | **1.6** | **0.8** | **1.2** | [1] |
| TBC1D7 | TBC1 domain family member 7 | **33.0** | **20.8** | **0.8** | [2] |
| PRKCZ | protein kinase C zeta | **17.7** | **9.1** | **1.3** | [2] |
| DLL3* | delta like canonical Notch ligand 3 | **11.9** | **0.5** | **5.3** | [1] |
| TRIM45 | tripartite motif containing 45 | **7.6** | **2.9** | **1.9** | [2] |
| FANK1 | fibronectin type III and ankyrin repeat domains 1 | **14.2** | **4.8** | **2.0** | [1],[2] |
| IP6K3 | inositol hexakisphosphate kinase 3 | **2.9** | **0.5** | **2.8** | [1],[2] |
| TCTN2 | tectonic family member 2 | **13.4** | **6.4** | **1.9** | [2] |
| WDR19 | WD repeat domain 19 | **25.4** | **16.5** | **0.6** | [2] |
| SLC34A3 | solute carrier family 34 member 3 | **0.5** | **0.3** | **1.1** | [1] |
| PFKP | phosphofructokinase, platelet | **122.7** | **41.8** | **1.6** | [3] |
| PCSK9 | proprotein convertase subtilisin/kexin type 9 | **0.5** | **0.1** | **2.2** | [2] |
| ATP5B | ATP synthase, H+ transporting, mitochondrial F1 complex, beta polypeptide | **469.0** | **362.2** | **0.4** | [3] |
| PAX2* | paired box 2 | **22.5** | **4.6** | **3.1** | [1] |
| CHCHD6 | coiled-coil-helix-coiled-coil-helix domain containing 6 | **9.1** | **6.0** | **1.1** | [2] |
| KIF1A | kinesin family member 1A | **74.9** | **45.6** | **0.8** | [3] |
| CABP1 | calcium binding protein 1 | **0.9** | **0.3** | **2.0** | known |
| EIF3F | eukaryotic translation initiation factor 3 subunit F | **31.6** | **20.5** | **0.6** | [3] |
| RPH3AL | rabphilin 3A-like (without C2 domains) | **0.7** | **0.3** | **1.8** | [1] |
| SLC16A5 | solute carrier family 16 member 5 | **9.5** | **3.8** | **1.6** | [1],[2] |
| MTMR11 | myotubularin related protein 11 | **37.6** | **24.1** | **0.7** | [2] |
| FCHO1 | FCH domain only 1 | **2.9** | **1.5** | **1.1** | [2] |
| MVP | major vault protein | **112.3** | **84.0** | **0.8** | [3] |
| LDB3 | LIM domain binding 3 | **1.1** | **0.2** | **2.6** | [2] |
| KCNH2* | potassium voltage-gated channel subfamily H member 2 | **1.6** | **0.7** | **1.6** | [1] |
| CBLN1* | cerebellin 1 precursor | **1.4** | **0.4** | **2.4** | [1],[2] |
| TMEM91* | transmembrane protein 91 | **17.7** | **3.3** | **2.6** | [1] |
| RIC3 | RIC3 acetylcholine receptor chaperone | **8.9** | **5.4** | **0.9** | [1] |
| WDR1 | WD repeat domain 1 | **175.7** | **125.9** | **0.8** | [3] |
| MCF2L | MCF.2 cell line derived transforming sequence like | **10.6** | **4.8** | **3.0** | [2] |
| ESRP2 | epithelial splicing regulatory protein 2 | **10.0** | **4.2** | **2.1** | [1] |
| ZFR2 | zinc finger RNA binding protein 2 | **1.7** | **0.6** | **1.6** | [2] |
| CLSTN3 | calsyntenin 3 | **27.8** | **16.0** | **0.9** | [1],[2] |
| GPR4* | G protein-coupled receptor 4 | **4.1** | **1.4** | **2.0** | [1],[2] |
| ACTN1 | actinin alpha 1 | **75.4** | **39.5** | **1.1** | [3] |
| CAMK2B | calcium/calmodulin dependent protein kinase II beta | **1.2** | **0.8** | **0.9** | [2] |
| MAPK8IP2 | mitogen-activated protein kinase 8 interacting protein 2 | **3.2** | **1.9** | **0.9** | [1] |
| TOX2 | TOX high mobility group box family member 2 | **2.4** | **0.8** | **3.3** | [1] |
| SLC27A2 | solute carrier family 27 member 2 | **3.7** | **1.4** | **1.8** | [1] |
| ACTG1 | actin gamma 1 | **2126.4** | **1422.2** | **0.8** | [3] |
| KCNH8 | potassium voltage-gated channel subfamily H member 8 | **1.2** | **0.6** | **1.3** | [1] |
| IFIT1 | interferon induced protein with tetratricopeptide repeats 1 | **217.0** | **30.2** | **3.9** | [2] |
| ABCC8 | ATP binding cassette subfamily C member 8 | **10.5** | **0.7** | **5.4** | [1],[2] |
| RAB25 | RAB25, member RAS oncogene family | **51.8** | **28.3** | **2.9** | [1],[2] |
| ADPRHL1 | ADP-ribosylhydrolase like 1 | **2.0** | **0.8** | **2.0** | [2] |
| PSMD3 | proteasome 26S subunit, non-ATPase 3 | **59.9** | **42.9** | **0.6** | [3] |
| GAL | galanin and GMAP prepropeptide | **18.9** | **3.3** | **2.6** | [2] |
| ATP2A3 | ATPase sarcoplasmic/endoplasmic reticulum Ca2+ transporting 3 | **10.0** | **3.6** | **1.8** | [2] |
| SCG3 | secretogranin III | **0.5** | **0.3** | **1.2** | [2] |
| STRC* | stereocilin | **1.7** | **0.3** | **3.6** | [1],[2] |
| CYB561* | cytochrome b561 | **31.5** | **19.2** | **0.9** | [1] |
| CCDC151 | coiled-coil domain containing 151 | **3.3** | **0.8** | **2.0** | [2] |
| HES6* | hes family bHLH transcription factor 6 | **50.7** | **6.9** | **3.1** | [1] |
| VWA5B2* | von Willebrand factor A domain containing 5B2 | **3.1** | **0.7** | **2.2** | [1],[2] |
| PAWR | pro-apoptotic WT1 regulator | **19.9** | **10.1** | **1.1** | [2] |
| AKNAD1 | AKNA domain containing 1 | **1.7** | **0.7** | **1.3** | [1] |
| TBC1D2 | TBC1 domain family member 2 | **14.7** | **7.5** | **1.3** | [1] |
| SLC8A2* | solute carrier family 8 member A2 | **1.8** | **0.6** | **1.7** | [1] |
| CCDC88B | coiled-coil domain containing 88B | **13.6** | **6.4** | **1.2** | [1] |
| TMEM30B | transmembrane protein 30B | **26.6** | **13.4** | **2.0** | [2] |
| AIG1 | androgen induced 1 | **65.8** | **41.5** | **0.8** | [2] |
| HSPA2 | heat shock protein family A (Hsp70) member 2 | **87.4** | **30.8** | **3.0** | [2] |
| NAT14 | N-acetyltransferase 14 (putative) | **15.4** | **11.4** | **0.5** | [2] |
| SCN1B* | sodium voltage-gated channel beta subunit 1 | **23.4** | **13.5** | **1.3** | [1] |
| FN3K | fructosamine 3 kinase | **23.0** | **13.1** | **0.9** | [1],[2] |
| BAIAP2L2* | BAI1 associated protein 2 like 2 | **3.6** | **2.4** | **0.8** | {1] |
| KNDC1 | kinase non-catalytic C-lobe domain containing 1 | **2.5** | **0.7** | **2.1** | {1] |
| TKT | transketolase | **148.6** | **99.1** | **0.7** | [3] |
| ARHGDIA | Rho GDP dissociation inhibitor alpha | **144.0** | **114.1** | **0.3** | [3] |
| PNMA2* | paraneoplastic Ma antigen 2 | **1.4** | **0.5** | **2.1** | [1] |
| NEURL1B* | neuralized E3 ubiquitin protein ligase 1B | **1.1** | **0.6** | **1.1** | [1] |
| ANP32A | acidic nuclear phosphoprotein 32 family member A | **63.9** | **49.2** | **0.4** | [3] |
| GALNT14 | polypeptide N-acetylgalactosaminyltransferase 14 | **5.1** | **2.0** | **2.0** | [1] |
| TTC24 | tetratricopeptide repeat domain 24 | **0.7** | **0.1** | **2.7** | [1],[2] |
| SLC35F3 | solute carrier family 35 member F3 | **2.0** | **0.6** | **3.1** | [1] |
| NSFL1C | NSFL1 cofactor | **49.6** | **33.8** | **0.6** | [3] |
| OTUD3* | OTU deubiquitinase 3 | **6.3** | **4.0** | **0.7** | [1],[2] |
| DNALI1 | dynein axonemal light intermediate chain 1 | **13.5** | **7.2** | **1.0** | [1],[2] |
| RAB3A | RAB3A, member RAS oncogene family | **4.3** | **1.5** | **1.6** | [1] |
| MAST1* | microtubule associated serine/threonine kinase 1 | **5.3** | **0.9** | **2.9** | [1] |
| ST3GAL5 | ST3 beta-galactoside alpha-2,3-sialyltransferase 5 | **16.6** | **7.3** | **1.7** | [2] |
| ESPNL* | espin-like | **0.7** | **0.3** | **1.3** | [1],[2] |
| FBXO15 | F-box protein 15 | **3.4** | **0.9** | **2.2** | [2] |
| DISP2* | dispatched RND transporter family member 2 | **1.8** | **0.5** | **2.5** | [1],[2] |
| AARS | alanyl-tRNA synthetase | **76.7** | **47.9** | **0.7** | [3] |
| TSPAN2* | tetraspanin 2 | **1.1** | **0.4** | **2.3** | [1],[2] |
| MORN1 | MORN repeat containing 1 | **4.2** | **3.1** | **0.6** | [2] |
| FUCA1 | fucosidase, alpha-L- 1, tissue | **34.4** | **26.1** | **0.5** | [3] |
| ELFN1* | extracellular leucine rich repeat and fibronectin type III domain containing 1 | **1.3** | **0.3** | **2.3** | [1],[2] |
| AGBL2 | ATP/GTP binding protein like 2 | **4.9** | **1.5** | **2.3** | [2] |
| AMPH | amphiphysin | **3.1** | **0.7** | **2.4** | [2],[3] |
| CASC1 | cancer susceptibility candidate 1 | **1.1** | **0.4** | **1.4** | [2] |
| ARTN | artemin | **16.8** | **5.5** | **1.7** | [1],[2] |
| B3GNT4 | UDP-GlcNAc:betaGal beta-1,3-N-acetylglucosaminyltransferase 4 | **4.0** | **1.8** | **1.3** | [1],[2] |
| PACSIN1* | protein kinase C and casein kinase substrate in neurons 1 | **1.0** | **0.3** | **2.1** | [1],[3] |
| PPFIA3 | PTPRF interacting protein alpha 3 | **3.7** | **2.3** | **0.8** | [1] |
| DDB1 | damage specific DNA binding protein 1 | **75.9** | **68.1** | **0.2** | [3] |
| CRTAM | cytotoxic and regulatory T-cell molecule | **0.7** | **0.1** | **2.6** | [2] |
| ECEL1* | endothelin converting enzyme like 1 | **1.2** | **0.3** | **2.3** | [1] |
| LRRC43 | leucine rich repeat containing 43 | **1.8** | **0.7** | **1.3** | [1],[2] |
| SYT17 | synaptotagmin 17 | **7.1** | **2.4** | **1.9** | [1] |
| CLEC18A | C-type lectin domain family 18 member A | **2.4** | **0.1** | **4.3** | [1],[2] |
| BBS1* | Bardet-Biedl syndrome 1 | **19.4** | **16.0** | **0.4** | [1],[2] |
| LUC7L2 | LUC7 like 2, pre-mRNA splicing factor | **32.0** | **25.9** | **0.3** | [3] |
| CIB2* | calcium and integrin binding family member 2 | **12.3** | **5.5** | **1.2** | [1],[2] |
| NMNAT3 | nicotinamide nucleotide adenylyltransferase 3 | **7.5** | **4.3** | **0.9** | [1],[2] |
| ADCY8 | adenylate cyclase 8 | **0.5** | **0.4** | **0.7** | [1],[2] |
| TTC39B* | tetratricopeptide repeat domain 39B | **3.8** | **2.2** | **1.0** | [1],[2] |
| ACSS2* | acyl-CoA synthetase short-chain family member 2 | **25.1** | **20.0** | **0.4** | [1],[2] |
| AP3M2 | adaptor related protein complex 3 mu 2 subunit | **12.7** | **11.5** | **0.2** | [2],[3] |
| NHLH1* | nescient helix-loop-helix 1 | **1.1** | **0.6** | **0.9** | [1],[2] |
| RAB36 | RAB36, member RAS oncogene family | **3.9** | **2.0** | **1.0** | [2] |
| HSP90AB1 | heat shock protein 90 alpha family class B member 1 | **245.2** | **190.7** | **0.4** | [3] |
| MOGAT1 | monoacylglycerol O-acyltransferase 1 | **0.4** | **0.1** | **2.1** | [1],[2] |
| POU2AF1 | POU class 2 associating factor 1 | **0.5** | **0.1** | **2.0** | [1] |
| WNT7A | Wnt family member 7A | **4.0** | **1.1** | **3.3** | [1] |
| DSCAML1* | DS cell adhesion molecule like 1 | **1.5** | **0.2** | **2.9** | [1] |
| STARD10* | StAR related lipid transfer domain containing 10 | **100.3** | **71.5** | **0.6** | [1][2],[3] |
| DLEC1 | deleted in lung and esophageal cancer 1 | **1.3** | **0.4** | **1.9** | [1] |
| RASD2* | RASD family member 2 | **6.9** | **3.4** | **1.1** | [1],[2] |
| REM2 | RRAD and GEM like GTPase 2 | **1.5** | **0.5** | **2.3** | [2] |
| ILDR1 | immunoglobulin like domain containing receptor 1 | **6.8** | **4.0** | **2.1** | [1],[2] |
| LFNG* | LFNG O-fucosylpeptide 3-beta-N-acetylglucosaminyltransferase | **4.9** | **1.0** | **2.5** | [1] |
| LMO1* | LIM domain only 1 | **0.6** | **0.2** | **2.3** | [1],[2] |
| NOD2 | nucleotide binding oligomerization domain containing 2 | **0.9** | **0.4** | **1.4** | [1],[2] |
| CYP17A1 | cytochrome P450 family 17 subfamily A member 1 | **5.4** | **0.6** | **4.2** | [2] |
| XPNPEP1 | X-prolyl aminopeptidase 1 | **50.2** | **39.5** | **0.4** | [3] |
| TDRD5 | tudor domain containing 5 | **0.7** | **0.4** | **1.8** | [1] |
| EMX2 | empty spiracles homeobox 2 | **3.4** | **1.8** | **1.0** | [1] |
| CPLX1* | complexin 1 | **2.6** | **1.0** | **1.6** | [1] |
| RIBC2 | RIB43A domain with coiled-coils 2 | **0.4** | **0.2** | **1.5** | [2] |
| YARS | tyrosyl-tRNA synthetase | **67.7** | **54.8** | **0.4** | [3] |
| MYO1C | myosin IC | **58.9** | **49.8** | **0.3** | [3] |
| SEMA6B | semaphorin 6B | **8.5** | **5.3** | **0.7** | [2] |
| CKB | creatine kinase B | **114.7** | **35.4** | **1.9** | [3] |
| SRRM4* | serine/arginine repetitive matrix 4 | **0.5** | **0.2** | **1.6** | [1] |
| EDN2 | endothelin 2 | **2.6** | **0.7** | **3.5** | [1] |
| GAS6* | growth arrest specific 6 | **30.5** | **12.4** | **2.7** | [1] |
| WWC1 | WW and C2 domain containing 1 | **45.9** | **24.7** | **2.0** | [1],[2] |
| CACNA2D2 | calcium voltage-gated channel auxiliary subunit alpha2delta 2 | **3.1** | **1.2** | **1.8** | [3] |
| SYT13* | synaptotagmin 13 | **29.6** | **9.3** | **3.7** | [1] |
| ARHGDIG* | Rho GDP dissociation inhibitor gamma | **1.5** | **0.9** | **1.0** | [1],[2] |
| PKHD1L1 | polycystic kidney and hepatic disease 1 (autosomal recessive)-like 1 | **1.1** | **0.2** | **2.8** | [1] |
| CD164L2* | CD164 molecule like 2 | **10.3** | **3.0** | **5.2** | [1],[2] |
| PGK1 | phosphoglycerate kinase 1 | **657.3** | **239.7** | **1.9** | [3] |
| OXTR | oxytocin receptor | **5.2** | **1.2** | **3.6** | [1] |
| EEF2 | eukaryotic translation elongation factor 2 | **473.0** | **355.9** | **0.4** | [3] |
| HERC3 | HECT and RLD domain containing E3 ubiquitin protein ligase 3 | **16.8** | **14.9** | **0.2** | [2] |
| GOT1 | glutamic-oxaloacetic transaminase 1 | **67.6** | **40.1** | **0.8** | [3] |
| CASZ1* | castor zinc finger 1 | **4.9** | **2.6** | **1.3** | [1],[2] |
| AGBL4 | ATP/GTP binding protein like 4 | **0.9** | **0.2** | **2.7** | [2] |
| EIF3B | eukaryotic translation initiation factor 3 subunit B | **79.2** | **54.8** | **0.6** | [3] |
| CRTAC1 | cartilage acidic protein 1 | **6.3** | **1.3** | **2.7** | [2] |
| GAPDH | glyceraldehyde-3-phosphate dehydrogenase | **2028.0** | **989.0** | **1.2** | [3] |
| UBXN10 | UBX domain protein 10 | **1.6** | **0.7** | **2.7** | [1],[2] |
| RBM24* | RNA binding motif protein 24 | **4.7** | **2.2** | **1.2** | [1] |
| PGAM1 | phosphoglycerate mutase 1 | **47.2** | **25.8** | **0.9** | [3] |
| BARHL1 | BarH like homeobox 1 | **0.4** | **0.1** | **1.9** | [1],[2] |
| RIN1* | Ras and Rab interactor 1 | **7.1** | **3.6** | **1.1** | [1] |
| NME3 | NME/NM23 nucleoside diphosphate kinase 3 | **28.3** | **17.0** | **0.8** | [3] |
| TMC1 | transmembrane channel like 1 | **1.3** | **0.5** | **2.5** | [1],[2] |
| SLC25A6 | solute carrier family 25 member 6 | **147.4** | **109.6** | **0.5** | [3] |
| HK2 | hexokinase 2 | **31.3** | **13.8** | **1.2** | [3] |
| KCNE2 | potassium voltage-gated channel subfamily E regulatory subunit 2 | **2.6** | **1.0** | **2.4** | [2] |
| NECAB2* | N-terminal EF-hand calcium binding protein 2 | **2.6** | **1.4** | **1.3** | [1] |
| STK32C | serine/threonine kinase 32C | **6.1** | **4.7** | **0.4** | [2] |
| TESC* | tescalcin | **31.9** | **16.4** | **2.3** | [1] |
| FHAD1 | forkhead associated phosphopeptide binding domain 1 | **3.7** | **1.7** | **1.2** | [2] |
| POU4F2 | POU class 4 homeobox 2 | **2.4** | **0.6** | **3.4** | [2] |
| TPPP | tubulin polymerization promoting protein | **1.0** | **0.6** | **1.2** | [1],[2] |
| RPS16 | ribosomal protein S16 | **717.2** | **559.3** | **0.4** | [3] |
| CYB561D2 | cytochrome b561 family member D2 | **4.1** | **1.7** | **1.5** | [2] |
| CLRN1 | clarin 1 | **0.8** | **0.4** | **2.0** | [2] |
| HSD17B14 | hydroxysteroid 17-beta dehydrogenase 14 | **21.3** | **12.7** | **0.8** | [2] |
| RASSF10 | Ras association domain family member 10 | **1.1** | **0.4** | **2.0** | [1],[2] |
| DDO | D-aspartate oxidase | **9.4** | **4.1** | **2.3** | [2] |
| ARPC4 | actin related protein 2/3 complex subunit 4 | **77.4** | **64.2** | **0.3** | [3] |
| STAC | SH3 and cysteine rich domain | **36.1** | **12.9** | **3.5** | [1],[2] |
| CEL | carboxyl ester lipase | **3.8** | **1.3** | **2.4** | [1] |
| ACAT2 | acetyl-CoA acetyltransferase 2 | **9.9** | **7.7** | **0.4** | [3] |
| AACS | acetoacetyl-CoA synthetase | **13.7** | **8.9** | **0.8** | [2] |
| SSTR2 | somatostatin receptor 2 | **0.9** | **0.3** | **1.5** | [1] |
| EVL | Enah/Vasp-like | **12.1** | **10.4** | **0.3** | [2] |
| LHX4 | LIM homeobox 4 | **0.5** | **0.3** | **0.5** | [1] |
| PTPRN2 | protein tyrosine phosphatase, receptor type N2 | **1.9** | **1.0** | **1.0** | [2] |
| CRB3 | crumbs 3, cell polarity complex component | **33.7** | **16.9** | **2.7** | [1],[2] |
| RAB3IP* | RAB3A interacting protein | **30.0** | **23.0** | **0.4** | [1],[2] |
| C21orf33 | chromosome 21 open reading frame 33 | **1.4** | **0.5** | **3.3** | [3] |
| PA2G4 | proliferation-associated 2G4 | **22.8** | **19.2** | **0.3** | [3] |
| MYL3 | myosin light chain 3 | **0.5** | **0.1** | **2.2** | [1],[2] |
| TPI1 | triosephosphate isomerase 1 | **563.7** | **248.6** | **1.5** | [3] |
| GNG8 | G protein subunit gamma 8 | **0.8** | **0.1** | **2.9** | [1] |
| IMPDH2 | inosine monophosphate dehydrogenase 2 | **122.2** | **104.1** | **0.2** | [3] |
| HNRNPA3 | heterogeneous nuclear ribonucleoprotein A3 | **18.0** | **14.6** | **0.3** | [3] |
| TRPM2 | transient receptor potential cation channel subfamily M member 2 | **1.5** | **1.2** | **0.3** | [1],[2] |
| SPAG8 | sperm associated antigen 8 | **2.6** | **1.4** | **0.9** | [1] |
| CLDN9 | claudin 9 | **41.3** | **20.3** | **2.7** | [1],[2] |
| PGAM2* | phosphoglycerate mutase 2 | **2.9** | **0.7** | **3.5** | [1],[2] |
| TMEM130 | transmembrane protein 130 | **0.7** | **0.3** | **1.9** | [1],[2] |
| PACRG | PARK2 coregulated | **6.3** | **2.0** | **2.5** | [1],[2] |
| EHD3 | EH domain containing 3 | **25.6** | **16.3** | **0.8** | [3] |
| BLNK | B-cell linker | **1.0** | **0.7** | **0.7** | [1] |
| CPEB3 | cytoplasmic polyadenylation element binding protein 3 | **1.5** | **0.8** | **1.0** | [2] |
| ABCC1* | ATP binding cassette subfamily C member 1 | **0.7** | **0.5** | **0.5** | [1] |
| ENO1 | enolase 1 | **1505.3** | **594.4** | **1.8** | [3] |
| FSCN1 | fascin actin-bundling protein 1 | **26.0** | **9.9** | **1.9** | [3] |
| CHGB* | chromogranin B | **1.6** | **0.9** | **1.1** | [1],[2] |
| SRSF1 | serine and arginine rich splicing factor 1 | **105.0** | **70.6** | **0.6** | [3] |
| FMN1 | formin 1 | **0.9** | **0.6** | **0.7** | [2] |
| PTGIR* | prostaglandin I2 (prostacyclin) receptor (IP) | **0.3** | **0.2** | **1.0** | [1] |
| SEMA7A* | semaphorin 7A (John Milton Hagen blood group) | **21.1** | **17.9** | **0.3** | [1] |
| RPS3 | ribosomal protein S3 | **462.5** | **341.4** | **0.5** | [3] |
| DLL4* | delta like canonical Notch ligand 4 | **7.0** | **2.1** | **3.3** | [1],[2] |
| TEKT2 | tektin 2 | **0.9** | **0.5** | **2.0** | [2] |
| ENO4 | enolase family member 4 | **0.3** | **0.2** | **1.1** | [1] |
| SH3GL2 | SH3 domain containing GRB2 like 2, endophilin A1 | **8.2** | **4.6** | **2.1** | [2] |
| OSCP1 | organic solute carrier partner 1 | **9.8** | **7.2** | **0.6** | [2] |
| GNMT* | glycine N-methyltransferase | **0.4** | **0.3** | **1.2** | [1],[2] |
| TEKT1 | tektin 1 | **0.2** | **0.2** | **0.7** | [1],[2] |
| TUBB4B | tubulin beta 4B class IVb | **318.7** | **212.2** | **0.8** | [3] |
| IRX2 | iroquois homeobox 2 | **6.2** | **2.7** | **1.5** | [2] |
| DYX1C1 | dyslexia susceptibility 1 candidate 1 | **2.1** | **1.0** | **3.0** | [2] |
| PCP4* | Purkinje cell protein 4 | **28.5** | **9.1** | **3.0** | [1],[2] |
| RSPH1 | radial spoke head 1 homolog | **2.4** | **1.5** | **1.0** | [1],[2] |
| RPRM | reprimo, TP53 dependent G2 arrest mediator candidate | **5.3** | **2.5** | **1.9** | [1] |
| FAM19A3* | family with sequence similarity 19 member A3, C-C motif chemokine like | **3.6** | **1.9** | **1.5** | [1],[2] |
| FBXO16 | F-box protein 16 | **6.9** | **3.2** | **2.2** | [1],[2] |
| TMPRSS6 | transmembrane protease, serine 6 | **4.3** | **2.0** | **3.1** | [2] |
| LDHA | lactate dehydrogenase A | **804.7** | **409.0** | **1.3** | [3] |
| RRP12 | ribosomal RNA processing 12 homolog | **17.9** | **12.7** | **0.5** | [2] |
| ARF1 | ADP ribosylation factor 1 | **220.8** | **194.9** | **0.2** | [3] |
| RSPH4A | radial spoke head 4 homolog A | **0.7** | **0.4** | **1.1** | [1],[2] |
| FAM83A | family with sequence similarity 83 member A | **0.2** | **0.1** | **1.0** | [1] |
| CACNA1D | calcium voltage-gated channel subunit alpha1 D | **8.1** | **3.4** | **2.5** | [2] |
| SP110 | SP110 nuclear body protein | **21.3** | **11.9** | **1.1** | [2] |
| TOM1 | target of myb1 membrane trafficking protein | **40.4** | **30.3** | **0.5** | [3] |
| LRRC10B | leucine rich repeat containing 10B | **1.1** | **0.6** | **1.2** | [1] |
| SNCG | synuclein gamma | **2.6** | **0.9** | **1.6** | [2] |
| EIF3D | eukaryotic translation initiation factor 3 subunit D | **123.6** | **105.6** | **0.2** | [3] |
| PRR15L* | proline rich 15 like | **4.6** | **2.2** | **3.1** | [1] |
| INPP5J | inositol polyphosphate-5-phosphatase J | **3.5** | **2.1** | **1.1** | [1] |
| LHX3* | LIM homeobox 3 | **0.7** | **0.5** | **0.7** | [1],[2] |
| BTN2A2 | butyrophilin subfamily 2 member A2 | **13.3** | **9.8** | **0.5** | [2] |
| PCP4L1 | Purkinje cell protein 4 like 1 | **8.4** | **2.7** | **2.3** | [1] |
| MYO3B* | myosin IIIB | **3.0** | **1.5** | **3.0** | [1],[2] |
| LRRC26 | leucine rich repeat containing 26 | **0.1** | **0.1** | **0.4** | [1] |
| AK7 | adenylate kinase 7 | **3.2** | **1.9** | **1.1** | [2] |
| FOXJ1* | forkhead box J1 | **2.2** | **0.9** | **2.4** | [1],[2] |
| CA2 | carbonic anhydrase 2 | **83.6** | **41.0** | **1.9** | [3] |
| SUMO2 | small ubiquitin-like modifier 2 | **38.6** | **34.2** | **0.2** | [3] |
| GRP | gastrin releasing peptide | **0.4** | **0.2** | **1.7** | [1],[2] |
| HECW1* | HECT, C2 and WW domain containing E3 ubiquitin protein ligase 1 | **0.1** | **0.1** | **0.2** | [1],[2] |
| ALB | albumin | **1.3** | **0.6** | **1.8** | [3] |
| CALML4 | calmodulin like 4 | **14.0** | **9.2** | **2.4** | [2] |
| ATP11A* | ATPase phospholipid transporting 11A | **0.2** | **0.1** | **0.6** | [1],[2] |
| CPE | carboxypeptidase E | **18.0** | **15.8** | **0.2** | [2] |
| PIP4K2A | phosphatidylinositol-5-phosphate 4-kinase type 2 alpha | **0.1** | **0.1** | **0.0** | [2] |
